# Insights into the Structure of the Spruce Budworm (*Choristoneura fumiferana*) Genome, as Revealed by Molecular Cytogenetic Analyses and a High-Density Linkage Map

**DOI:** 10.1534/g3.118.200263

**Published:** 2018-06-27

**Authors:** Sandrine Picq, Lisa Lumley, Jindra Šíchová, Jérôme Laroche, Esther Pouliot, Bryan M. T. Brunet, Roger C. Levesque, Felix A. H. Sperling, František Marec, Michel Cusson

**Affiliations:** * Laurentian Forestry Centre, Natural Resources Canada, Quebec City, Quebec, Canada, G1V 4C7; † Institut de Biologie Intégrative et des Systèmes (IBIS), Université Laval, Quebec City, Quebec, Canada, G1V 0A6; ‡ Royal Alberta Museum, Edmonton, Alberta, Canada, T5J 0G2; § Biology Centre, Czech Academy of Sciences, Institute of Entomology, 370 05 České Budějovice, Czech Republic; ** Department of Biological Sciences, Biosciences Centre, University of Alberta, Edmonton, Alberta, Canada, T6G 2R3

**Keywords:** *Choristoneura fumiferana*, linkage map, genotyping-by-sequencing, karyotype, neo-Z chromosome

## Abstract

Genome structure characterization can contribute to a better understanding of processes such as adaptation, speciation, and karyotype evolution, and can provide useful information for refining genome assemblies. We studied the genome of an important North American boreal forest pest, the spruce budworm, *Choristoneura fumiferana*, through a combination of molecular cytogenetic analyses and construction of a high-density linkage map based on single nucleotide polymorphism (SNP) markers obtained through a genotyping-by-sequencing (GBS) approach. Cytogenetic analyses using fluorescence *in situ* hybridization methods confirmed the haploid chromosome number of n = 30 in both sexes of *C. fumiferana* and showed, for the first time, that this species has a WZ/ZZ sex chromosome system. Synteny analysis based on a comparison of the *Bombyx mori* genome and the *C. fumiferana* linkage map revealed the presence of a neo-Z chromosome in the latter species, as previously reported for other tortricid moths. In this neo-Z chromosome, we detected an *ABC transporter C2* (*ABCC2*) gene that has been associated with insecticide resistance. Sex-linkage of the *ABCC2* gene provides a genomic context favorable to selection and rapid spread of resistance against *Bacillus thuringiensis* serotype *kurstaki* (Btk), the main insecticide used in Canada to control spruce budworm populations. Ultimately, the linkage map we developed, which comprises 3586 SNP markers distributed over 30 linkage groups for a total length of 1720.41 cM, will be a valuable tool for refining our draft assembly of the spruce budworm genome.

The order Lepidoptera (moths and butterflies) is one of the most diverse taxa of insects, with at least 157,000 species described to date (Kristensen *et al.* 2007; van Nieukerken *et al.* 2011). Among these species, many have been extensively studied due to their pest status, whereas others have been used as experimental models in fields as diverse as physiology, genetics, evolution and ecology (Roe *et al.* 2010). For example, the diamondback moth, *Plutella xylostella*, has been used in many studies examining the development of insecticide resistance (You *et al.* 2013), while the squinting bush brown, *Bicyclus anynana*, whose wing color patterns vary depending on the season, has proven a valuable system to study the evolution of phenotypic plasticity (Brakefield *et al.* 2007). Genomic characteristics unique to Lepidoptera have also drawn much attention. Moths and butterflies have holokinetic chromosomes (*i.e.*, chromosome lacking a primary constriction, the centromere; Marec *et al.* 2010; Sahara *et al.* 2012) and meiotic recombination is limited to the male sex (Suomalainen *et al.* 1973; Turner and Sheppard 1975; Traut 1977; Nokkala 1987; Traut *et al.* 2007). Moreover, lepidopteran chromosome numbers show a high degree of variability, ranging from n = 5 to n = 224-226 in the species examined (Marec *et al.* 2010; Lukhtanov 2015; Blackmon *et al.* 2017), with an average around the inferred ancestral number of n = 31 chromosomes (Suomalainen 1969; Lukhtanov 2000; Ahola *et al.* 2014; Yasukochi *et al.* 2016), found in *ca*. one-third of species (Suomalainen 1969; Robinson 1971; De Prins and Saitoh 2003; Blackmon *et al.* 2017). Interestingly, a high degree of gene synteny has been observed among homologous chromosomes across distantly related taxa (Pringle *et al.* 2007; Yasukochi *et al.* 2009; Baxter *et al.* 2011; Yoshido *et al.* 2011; Van’t Hof *et al.* 2013; Ahola *et al.* 2014), and the patterns of synteny described so far indicate that the numerically altered karyotypes evolved via fusion and/or fission events (Marec *et al.* 2010; Ahola *et al.* 2014; Šíchová *et al.* 2015). Finally, the order Lepidoptera, along with its sister group Trichoptera (caddisflies) and some tephritid fruit flies, is part of a restricted assemblage of insects in which the female is the heterogametic sex (ZW or Z0) (Bush 1966; Traut *et al.* 2007; Marec *et al.* 2010; Sahara *et al.* 2012; Blackmon *et al.* 2017).

The spruce budworm, *Choristoneura fumiferana*, is another lepidopteran species that has attracted much research attention. Belonging to the family Tortricidae, it is native to North America, and its larvae mainly feed on the foliage of spruces and true firs. One of the hallmarks of spruce budworm population dynamics is the development of severe outbreaks lasting *ca*. 10 years and recurring every 30 to 40 years (Jardon *et al.* 2003; Boulanger *et al.* 2012). During these outbreaks, intense defoliation typically results in major growth reductions, and several years of severe infestations can cause widespread tree mortality (MacLean 1985). While considered a very serious pest of North American spruce and fir stands, the spruce budworm is also seen as playing a positive, essential role in forest renewal (MacLean 2016). Since 2006, a new outbreak has gradually developed and spread over several regions of the province of Quebec, affecting 7.1 MHa as of 2017 (Ministère des forêts, de la faune et des parcs 2017). Due to its significant economic impact on the pulp and lumber industry across the Canadian boreal forest, the spruce budworm has been the subject of numerous studies and has imposed itself as a model for the study of insect population dynamics in North America (Pureswaran *et al.* 2016).

The spruce budworm has also been at the center of many studies examining various aspects of its biology, including its cytogenetic characteristics. It was reported that its karyotype consists of 30 pairs of chromosomes, including a pair of large chromosomes, presumably sex chromosomes (Ennis 1976; Harvey 1997). According to recent data, large sex chromosomes are a typical feature of most tortricids, with Z chromosomes having arisen from the fusion between an ancestral Z chromosome and an autosome corresponding to chromosome 15 of *Bombyx mori* (Nguyen *et al.* 2013; Šíchová *et al.* 2013). However, the occurrence of a similar fusion in *C. fumiferana* has yet to be confirmed. In addition, the structure and composition of the W chromosome varies greatly among lepidopteran species, and characterization of these features can provide novel insights into the formation and evolution of sex chromosomes in the Lepidoptera (Traut and Marec 1997; Yoshido *et al.* 2005; Vítková *et al.* 2007; Šíchová *et al.* 2013; Dalíková *et al.* 2017; Mongue *et al.* 2017). In *C. fumiferana*, composition of the W chromosome has not yet been examined.

In the present work, we developed a SNP-based high-density linkage map of *C. fumiferana* and used cytogenetic techniques to study the composition and structure of its genome. More specifically, (i) we confirmed the presence of 30 pairs of chromosomes in both sexes of this species, including a large pair of sex chromosomes, (ii) we used synteny analysis to confirm the existence of a neo-Z chromosome and (iii) we conducted cytogenetic characterization of the structure of the W chromosome. The present work was initiated as part of a *C. fumiferana* genome project, whose main goal is to generate a high-quality reference genome for this species. The high-density linkage map developed here will help to position scaffolds on chromosomes.

## Materials and Methods

### Karyotype analysis

Chromosome number for *Choristoneura fumiferana* was determined from mitotic metaphase cells stained by fluorescence *in situ* hybridization (FISH) using (TTAGG)*_n_* telomeric probe (tel-FISH), which enables identification of chromosome ends. Mitotic chromosomes were obtained from wing imaginal discs of 5^th^ instar male and female larvae using the spreading technique described in Mediouni *et al.* (2004). The telomeric probe was generated by non-template PCR according to the protocol of Sahara *et al.* (1999) and labeled with Cy3-dUTP (Jena Bioscience, Jena, Germany) using a Nick Translation Kit (Abbott Molecular Inc., Des Plaines, USA) with 75 min incubation at 15°. FISH with the (TTAGG)*_n_* telomeric probe was performed following the protocol of Yoshido *et al.* (2005). The hybridization mixture contained 100 ng of telomeric probe and 25 µg of sonicated salmon sperm DNA (Sigma-Aldrich, St. Louis, USA) in 10 µL of 50% formamide and 10% dextran sulfate in 2 x SSC.

To identify the sex chromosomes of *C. fumiferana*, we used genomic *in situ* hybridization (GISH) combined with tel-FISH as described in Yoshido *et al.* (2005) and Šíchová *et al.* (2015). GISH is based on hybridization of a fluorescently labeled female genomic DNA (gDNA) in the presence of an excess of unlabeled competitor DNA (*i.e.*, male gDNA). With this approach, the W chromosome is identified by strong binding of female labeled DNA due to its repetitive character. This approach has successfully identified the W chromosome in several lepidopteran species (Mediouni *et al.* 2004; Fuková *et al.* 2005; Yoshido *et al.* 2006).

Spread chromosome preparations were obtained from testes and ovaries of 5^th^ instar larvae as described in Mediouni *et al.* (2004). For GISH, total gDNA from males and females was isolated using the CTAB method adapted from Winnepenninckx *et al.* (1993). Female gDNA was labeled with fluorescein-12-dUTP (Jena Bioscience) using the Nick Translation Kit (Abbott Molecular Inc.), with 4 h of incubation at 15°, and male gDNA was sonicated using a Sonopuls HD 2070 (Bandelin Electric, Berlin, Germany) and used as competitor DNA. For GISH combined with tel-FISH, the probe mixture contained fluorescein-labeled female gDNA (300 ng), Cy3-labeled telomeric probe (100 ng), sonicated male gDNA (3 μg) and sonicated salmon sperm DNA (25 μg).

Preparations for both FISH experiments were counterstained with 0.5 mg/mL DAPI, mounted in antifade based on DABCO (Sigma-Aldrich), and observed under a Zeiss Axioplan 2 microscope (Carl Zeiss Jena, Germany). Black-and-white images were recorded with a cooled monochrome CCD camera XM10 using cellSens Standard software, version 1.9 (Olympus Europa Holding, Hamburg, Germany). All images were captured separately for each fluorescent dye, pseudocolored (light blue for DAPI, green for fluorescein and red for Cy3) and superimposed with Adobe Photoshop, version 7.0.

### Insect material and crossing strategy for the linkage map

**Figure 1:**
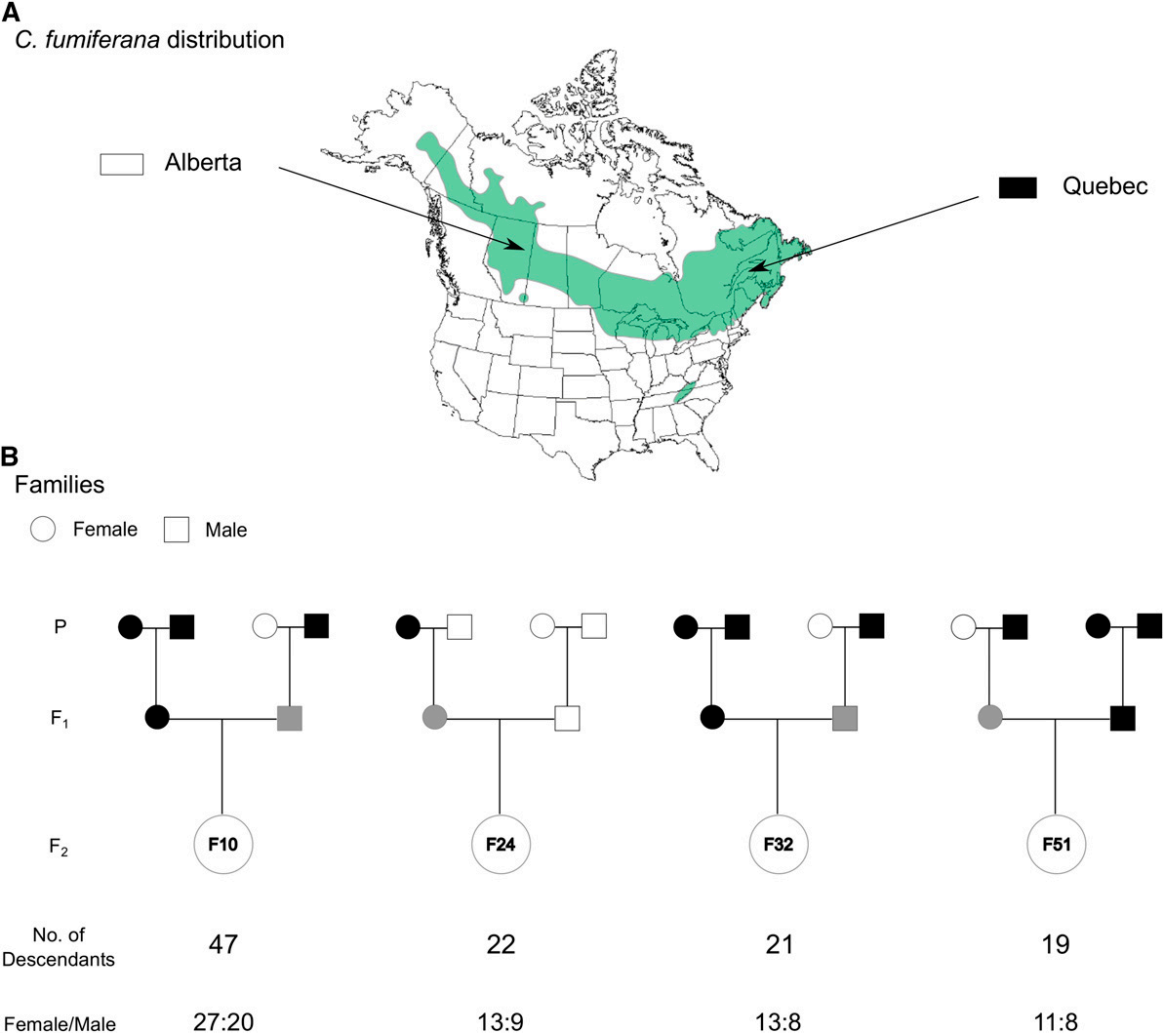
Sampling and crossing strategies employed to generate the *Choristoneura fumiferana* linkage map. (a) Map showing the geographic range of *C. fumiferana* in North America and the geographic origins of the two populations (Alberta and Quebec) used to generate the four families depicted below (adapted from Lumley and Sperling 2011). (b) Graphical representation of the family design used to generate the linkage map, with information on the number of descendants/family and the sex-ratio within each family. P, F1 and F2 labels identify, respectively, parent, first and second generations.

Parents were derived from *C. fumiferana* 4^th^-6^th^ instar larvae collected in 2013 from two Canadian populations in Alberta (AB; north of Conklin, 55.6985 N, -111.0841 W) and Quebec (QC; Gaspésie region, 48.4675 N, -68.1946 W). These two populations were selected on the basis of the large distance separating them (∼3000 km), each being near the longitudinal extremes of this species’ range in North America (Figure 1). We reasoned that genetic differentiation would likely be maximal between these two populations, thereby maximizing the number of informative loci between parents. Larvae were shipped to the Laurentian Forestry Centre (Quebec City, Canada) and reared to the adult stage. To obtain F_1_ families, we made 67 crosses according to the following combinations: QC male x QC female crosses (n = 20), QC male x AB female (n = 27), AB male x QC female (n = 12) and AB male x AB female (n = 8). Of these crosses, 39 were successful in producing progeny. This F_1_ generation was reared to the adult stage using rearing conditions favoring the bypassing of diapause (*i.e.*, exposure to continuous light); in nature, 2^nd^ instar spruce budworm larvae undergo winter diapause (*i.e.*, dormancy) for about eight months. These rearing conditions allowed us to expedite the process of obtaining linkage-map families. Then, 86 backcrosses were made to obtain the F_2_ generation (see details in File S1). For backcross progeny, we used standard rearing conditions (*i.e.*, with diapause) to achieve the highest rearing success possible. We successfully generated 24 F_2_ families, but most of them had fewer than 10 descendants. Given that substantial numbers of descendants tend to maximize the number of detectable recombination events used to build an informative linkage map, we selected the four largest unrelated F_2_ families available, representing 109 descendants in total (Figure 1). In addition, this multifamily mapping approach increased the probability of capturing segregating markers.

### DNA extraction and sequencing

Before DNA extraction, thoraces and legs of adult moths were frozen in liquid nitrogen and ground using a Retsch MM 200 mixer mill (Retsch technology, Haan, Germany). Then, DNA was extracted with the DNeasy 96 Blood & Tissue Kit (Qiagen, Carlsbad, CA, USA) following the manufacturer’s instructions, with the exception of an additional RNase A treatment before the addition of buffer AL/ethanol (4 µL of 100 mg/mL Rnase A; 5 min digestion at room temperature). DNA purity of the extracts was assessed using a NanoDrop 8000 spectrophotometer (Thermo scientific, Waltham, MA, USA), and DNA concentration was evaluated using Quant-iT PicoGreen dsDNA Assay Kit (Invitrogen, Carlsbad, CA, USA). Samples were diluted to 20 ng/μL prior to library construction.

To prepare a reduced-representation library for sequencing, we used a modified genotyping-by-sequencing (GBS) protocol in which two restriction enzymes (*Pst*I/*Msp*I) and a Y-adapter were employed (Mascher *et al.* 2013). To ensure sufficient read depth, the library was sequenced on three P1v3 chips using HiQ reagents on an Ion Proton sequencer (Thermo scientific, Waltham, MA, USA). GBS library construction and sequencing were carried out at the Plate-forme d’analyses génomiques of the Institut de Biologie Intégrative et des Systèmes (IBIS) at Université Laval (Quebec City, QC, Canada).

### SNP identification

Prior to analyses, read quality was assessed using the FastQC v. 0.11.1 software (Andrews 2016). SNP calling was carried out using both a reference-genome-independent method, *i.e.*, the *de novo* Universal Network Enabled Analysis Kit (UNEAK) pipeline (Lu *et al.* 2013), and the Fast-GBS pipeline, which maps reads to a reference genome (Torkamaneh *et al.* 2017). This dual SNP calling approach was chosen to minimize the loss of genetic variation due to missing regions in the reference genome (Lu *et al.* 2013). Indeed, at the time these analyses were conducted, our draft *C. fumiferana* genome (bw6 version, Cusson *et al.* unpublished data) was thought to have missing regions that may be accessible using the uneak pipeline. uneak was run with the following parameter settings: minimum tag count ***c*** = 5 (default), error tolerance rate in the network filter ***e*** = 0.03 (default), and minimum minor allele frequency mnMAF = 0.01. Briefly, uneak retains all reads containing a barcode and a restriction enzyme cut site, in addition to being devoid of missing data in the first 64 bp after the barcode. Reads are then clustered into tags (*i.e.*, reads displaying 100% identity), and only tags with ***c*** ≥ 5 are retained. Then, networks of tags differing by one bp are built. In these different networks, tags with read counts corresponding to 3% (e = 0.03) or less of read counts from the adjacent tags are considered errors. The edges connecting the “error” tags to “real” tags are then sheared, thus dividing networks into sub-networks or decreasing the number of tags present in networks. At the end of the process, only tag-pair networks are retained as potential SNPs; networks with multiple tags are discarded.

The procedure used by Fast-GBS for SNP identification first involves de-multiplexing and adapter trimming, keeping only reads ≥ 50 bp (user-defined). These reads are then mapped onto the reference genome, a step performed by the Burrows-Wheeler Aligner software package (BWA; Li and Durbin 2009) using the Maximal Exact match (MEM) algorithm. SNP calling is then carried out by Platypus v0.8.1 (Rimmer *et al.* 2014), using the following parameters: minimum number of supporting reads for a variant to be called (minReads) = 2, minimum read mapping quality (minMapQual) = 10, and minimum base-calling quality (minBaseQual) = 10.

### SNP filtering

Missing data and genotyping errors hinder the correct ordering of markers and cause expansion of the linkage map (Hackett and Broadfoot 2003; Cartwright *et al.* 2007). To avoid these biases, we discarded SNPs genotyped in < 80% of individuals, as well as those showing a minimum allele frequency < 0.01 and those displaying heterozygosity > 0.75. In addition, we filtered out SNPs showing significant deviation from Mendelian or sex chromosome (Z-linked) inheritance patterns (*P* < 0.01) in one family out of four (see Chen *et al.* 2014). The first SNP filtering steps were carried out using VCFtools (Danecek *et al.* 2011), whereas the Mendelian/sex chromosome inheritance filtering was conducted using an in-house R script.

### Linkage map construction

The linkage map was constructed using the Lep-MAP software (original version, Rastas *et al.* 2013). This software simultaneously analyzes thousands of markers from several outbred families and takes into account the absence of recombination in lepidopteran females (Robinson 1971; Suomalainen *et al.* 1973; Turner and Sheppard 1975). First, we employed the module *EstimateLODLimit* to choose the logarithm-of-odds (LOD) score threshold, which is used to determine whether or not markers belong to the same linkage group (LG). Based on 100 datasets randomly generated from the input data, an empirical distribution of maximum LOD scores is calculated from which the LOD value can be chosen with a desired significance level. Estimation of the LOD limit was made considering only maternal information (*maleprior* = -1). Then, assignment to LGs was carried out using the *SeparateChromosomes* module, on the basis of an LOD score limit of 6.8 (*p* value = 0.01) and considering only maternal information (*maleprior* = -1). At this step, only LGs with five or more markers were kept (*sizeLimit* = 5). Then, singular markers were added to the defined LGs, using the *JoinSingles* module with all informative markers and an LOD score limit of 8.4 (obtained by *EstimateLODLimit* with 7% significance and all informative markers). After the *JoinSingles* step, markers with more than 15 missing genotypes (13%) were removed from the LGs. Finally, ordering and positioning of markers was computed using the *OrderMarkers* module of Lep-MAP, with constant rates for genotype errors and recombination. For each LG, ten independent runs were carried out, at the end of which the marker ordering with the best likelihood was kept. LG-assigned markers with a genotype error rate estimate > 0.1 were then removed (genotype error rate estimation is given for each marker in the output of the *OrderMarkers* module). In addition, markers near the ends of LGs were also removed if they contributed to >1% of map length.

### Synteny with the Bombyx mori genome

Reads containing SNPs used in generating the linkage map were mapped onto the genome of the silkworm, *Bombyx mori* (KAIKObase v. 3.2.2, http://sgp.dna.affrc.go.jp/KAIKO), using tblastx analysis (e-score cut-off value of 1.0e-03). KAIKObase has the advantage of providing chromosome assignments for the scaffolds forming the genome assembly of *B. mori*. As a complement to the above analysis, we also conducted a tblastx analysis against the genome of the Glanville fritillary, *Melitaea cinxia* (Lepbase version, http://lepbase.org), which has n = 31 chromosomes; this number corresponds to what is considered to be the ancestral number of chromosomes for the Lepidoptera (Suomalainen 1969; Lukhtanov 2000; Ahola *et al.* 2014; Yasukochi *et al.* 2016), thus making *M. cinxia* a valuable subject for syntenic analysis. Scaffold chromosome assignment for *M. cinxia* was obtained from the website of the Glanville fritillary genome project (Metapopulation research center, 2017, https://www.helsinki.fi/en/beta/metapopulation-research-centre/downloads; last consulted July 3, 2018). A graphical representation of the results of our *C. fumiferana*–*B. mori* synteny analysis was generated using the R package *circlize* (Gu *et al.* 2014).

### Data availability

File S1 details the experimental design used to generate spruce budworm families for the linkage map. File S2 summarizes linkage map information *i.e.*, number of markers and size in cM, for each linkage group. File S2 also provides details on position, errors and sequence for each marker included in the linkage map, and the significant hits in tblastx searches against the *Bombyx mori* and the *Melitaea cinxia* genomes. File S3 details assignment of the *Choristoneura fumiferana* linkage groups to *B. mori* and the *M. cinxia* chromosomes. Supplemental material and SNP data are available at Figshare: https://doi.org/10.25387/g3.6686543.

## Results

### Karyotype analysis

**Figure 2:**
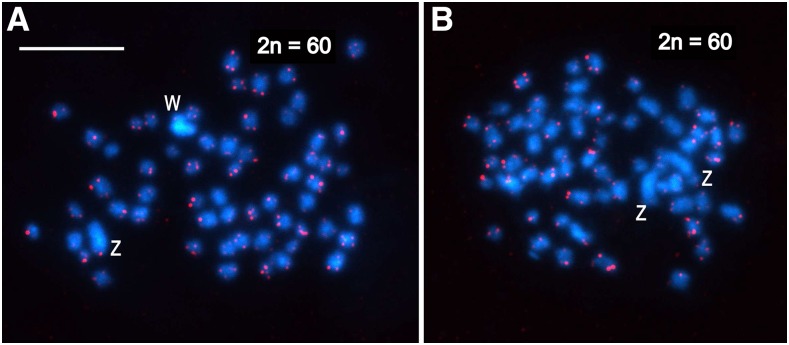
Karyotype analysis of mitotic chromosomes of *Choristoneura fumiferana* female (a) and male (b) by FISH using the (TTAGG)*_n_* telomeric probe. Hybridization signals of the Cy3-dUTP-labeled telomeric probe (red) indicate chromosome ends; chromosomes were counterstained with DAPI (blue). W and Z labels identify the largest chromosomes in mitotic complements, *i.e.*, the sex chromosomes. (a) Mitotic metaphase of *C. fumiferana* female with a heterochromatic W chromosome and a large Z chromosome (2n = 60). (b) Mitotic metaphase of *C. fumiferana* male with two large Z chromosomes (2n = 60). Scale bar = 10 μm.

**Figure 3:**
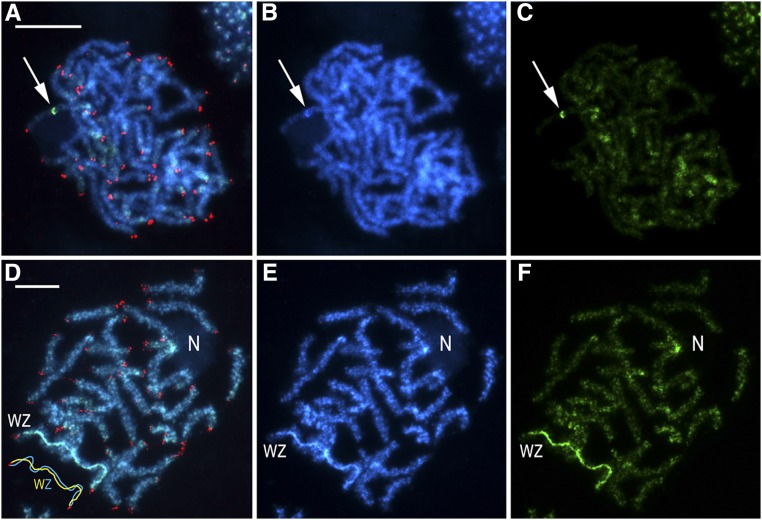
Genomic *in situ* hybridization combined with the (TTAGG)*_n_* telomeric probe in pachytene chromosomes in male (a-c) and female (d-f) *Choristoneura fumiferana*. Female-derived genomic probe was labeled with fluorescein-12-dUTP (green), and the telomeric probe with Cy3-dUTP (red); chromosomes were counterstained with DAPI (blue). Panels (a-c) show a male pachytene complement; arrows indicate heterochromatic block highlighted with the female genomic probe. Panels (d-f) show a female pachytene complement; “WZ” label identifies the sex chromosome bivalent (see schematic drawing in the lower left corner of panel d), where discrimination of the W chromosome is provided by the female-derived genomic probe; “N” indicates a nucleolus associated with a heterochromatic region (showing strong hybridization signals of the female genomic probe in panel d-f) of an autosome bivalent. (a, d) Merged images of preparations hybridized with female-derived genomic probe and telomeric probe, and counterstained with DAPI; (b, e) DAPI staining pattern; (c, f) hybridization pattern obtained using female-derived genomic probe. Scale bars = 10 μm.

The molecular cytogenetic analyses of mitotic chromosomes of *Choristoneura fumiferana* confirmed the presence of 2n = 60 chromosomes in both males and females (Figure 2), *i.e.*, the haploid chromosome number is n = 30. In both sexes, two chromosomes stood out by their larger size (Figure 2); in female preparations, one of these large chromosomes was more intensely stained with DAPI (blue) (Figure 2a; Figure 3e) and homogeneously highlighted with the green-labeled female gDNA probe (Figure 3d, f). These two large chromosomes represent the ZZ pair of sex chromosomes in males, and the WZ pair in females, where the intensely stained chromosome is the W chromosome composed of heterochromatin. Interestingly, in preparations of pachytene oocyte chromosomes stained by GISH and tel-FISH, the Z chromosome was clearly observed to wrap around the W chromosome to adjust its length for correct pairing; as a consequence, it appeared larger than its W counterpart (Figure 3d, e – see schematic representation). With GISH, strong binding of the female-derived genomic probe over the entire length of the W chromosome showed a high degree of molecular differentiation between the W and Z sex chromosomes (Figure 3d, f). The GISH analysis also highlighted a heterochromatic double-dot in one autosome bivalent in both sexes (Figure 3a-c; not shown for females) and an interstitial heterochromatic region of another autosome bivalent associated with the nucleolus (Figure 3d-f). This region obviously represents the nucleolus organizer region (NOR).

### SNP identification and filtering

The *de novo* UNEAK pipeline identified 208,098 polymorphic SNPs (Table 1), 1958 of which remained after eliminating those that did not genotype in at least 80% of individuals or did not present a minor allele frequency > 0.01. We then discarded an additional 679 SNPs that showed significant deviation from Mendelian or sex chromosome (Z-linked) inheritance patterns. In comparison, the reference-genome-based Fast-GBS pipeline generated 30,854 SNPs (SNPs having passed quality criteria in Platypus v0.8.1). Among these, we discarded markers that were not genotyped in at least 80% of individuals, presented a minor allele frequency < 0.01 or were triallelic. Of the remaining 22,094 SNPs, we discarded 9473 that showed significant deviation from Mendelian or sex chromosome (Z-linked) inheritance patterns. One descendant of family 24 (Figure 1) revealed Z-linked SNP segregation incompatible with the sex determined at the pupal stage; for the further analyses this individual was discarded. At the end of this filtering process, we were left with 13,900 SNPs, 12,621 and 1279 of which were identified using the Fast-GBS and UNEAK pipelines, respectively (Table1).

**Table 1:** Number of SNPs retained after each filtering step as a function of the SNP identification pipeline used

SNP filtering step	UNEAK		Fast-GBS	
	*Filtered SNPs*	*Remaining SNPs*	*Filtered SNPs*	*Remaining SNPs*
Pipeline output		208,098		30,854
Genotypes in > 80% of individuals, minor allele frequency > 0.01 and diallelic^1^	206,140	1958	8760	22,094
Deviation from mendelian or sex chromosome (Z-linked) segregation	679	1279	9473	12,621
Total SNPs used for the linkage map	**13,900**			

1 The diallelic filtering step was run only for the Fast-GBS pipeline; the UNEAK pipeline automatically discards non-diallelic SNPs.

### Construction of a Choristoneura fumiferana linkage map

We ran Lep-MAP on 13,900 SNPs, 7610 and 8012 of which were maternally and paternally informative, respectively (*i.e.*, heterozygous in the mother and the father of one of the crosses). The *SeparateChromosomes* module initially allocated 3180 SNPs to 30 LGs, while the *JoinSingles* module added 1853 SNPs to these 30 LGs, for a total of 5192 SNPs. After these two steps, 99 markers were removed from the LGs because more than 15 individuals presented missing genotypes (13%) for these markers. Then, the *OrderMarkers* module successfully ordered 3741 SNPs across 30 LGs. Among these 3741 SNPs, we discarded 50 markers presenting a genotype error estimate > 0.1 and 14 markers located near LG tips and contributing over 20 cM (>1.2%) to map length. In addition, we found 78 duplicated SNPs on the linkage map, a situation arising from our dual SNP identification approach (*i.e.*, UNEAK + Fast-GBS pipelines). In these cases, we retained the SNP copy presenting the least amount of missing data, typically the one identified using the Fast-GBS pipeline. Finally, close examination of the SNPs identified using the UNEAK pipeline indicated that 13 of them were in fact duplicates that escaped detection due to the presence of an indel within a mono-nucleotide repetitive region of both alleles of a locus (for details on this property of UNEAK, see Picq *et al.* 2018). Once again, the SNP copy presenting the least amount of missing data were retained.

**Figure 4:**
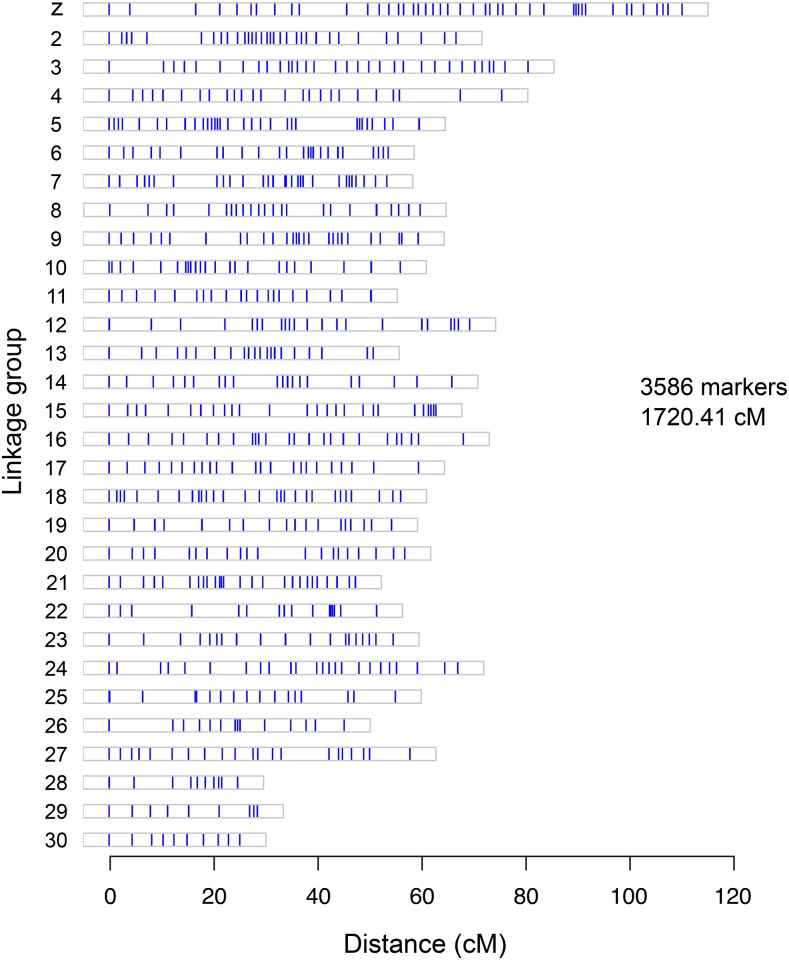
Linkage map generated for the spruce budworm, *Choristoneura fumiferana*, using the Lep-MAP software; based on 3586 SNP markers (vertical lines in each box). Linkage group numbering is derived from the Lep-MAP output.

In the end, the GBS/SNP-based linkage map we developed for *C. fumiferana* is made up of 3586 markers forming 30 linkage groups (LGs), for a total length of 1720.41 cM (Figure 4, File S2). The number of LGs found is in agreement with the number of chromosomes identified by our cytogenetic analyses. The genetic length of each LG ranges from 24.72 to 110.22 cM, with an average inter-locus distance varying between 0.31 and 1.23 cM among LGs, and a number of SNPs ranging from 22 to 352 per LG. The longest LG, with 352 SNPs along 110.22 cM, revealed a SNP segregation pattern characteristic of the Z chromosome (female offspring appear homozygous for one of the father’s alleles, indicating hemizygosity). LG numbering is that provided by the Lep-Map software. Among the 3586 markers used to generate the linkage map, 3394 (94.65%) were identified using the reference-genome-dependent Fast-GBS pipeline, while the 192 remaining SNPs (5.35%) were found using the *de novo* UNEAK pipeline; 35 of these SNPs were in regions absent from our reference genome, confirming an earlier assessment of the bw6 assembly’s completeness. Thus, combining the two SNP identification approaches used here proved to be both appropriate and efficient, as judged by the quality and density of markers found, making it possible to obtain the expected number of linkage groups.

It must be pointed out that the *SeparateChromosomes* module, parameterized with an LOD score limit of 6.8, identified a 31^st^ LG featuring 39 SNP markers. However, the *ordermarker* module could order only 9 of these markers. After removing three markers that displayed a genotype error estimate > 0.1, we ran again the *ordermarker* module on the remaining markers of the 31^st^ LG; this time, the *ordermarker* module was unable to order markers (blank output). To further explore this phenomenon, we ran the *SeparateChromosomes* module again, with a lower LOD score limit of 6.2. This time, the 39 SNP markers were added to the end of the 27^th^ LG. After a first run of the *ordermarker* module followed by the deletion of markers displaying a genotype error estimate > 0.1, the *ordermarker* module was run a second time on this elongated LG. In the end, only 6 markers out of 39 could be successfully ordered, but they were 40 cM away from the nearest marker on LG 27 and spanned 91.41 cM. With these 6 additional markers, the LG 27 doubles in size. Eventually, the sequences associated with these 6 ordered markers did not generate significant hits against the *B. mori* genome or against the NCBI nr database (TBLASTX analyses, expected value cut-off: 1.0e- 03). In light of these results, we discarded the 31^st^ LG obtained with the *SeparateChromosomes* module, parameterized with an LOD score limit of 6.8.

### Chromosomal homology through synteny analysis

**Figure 5:**
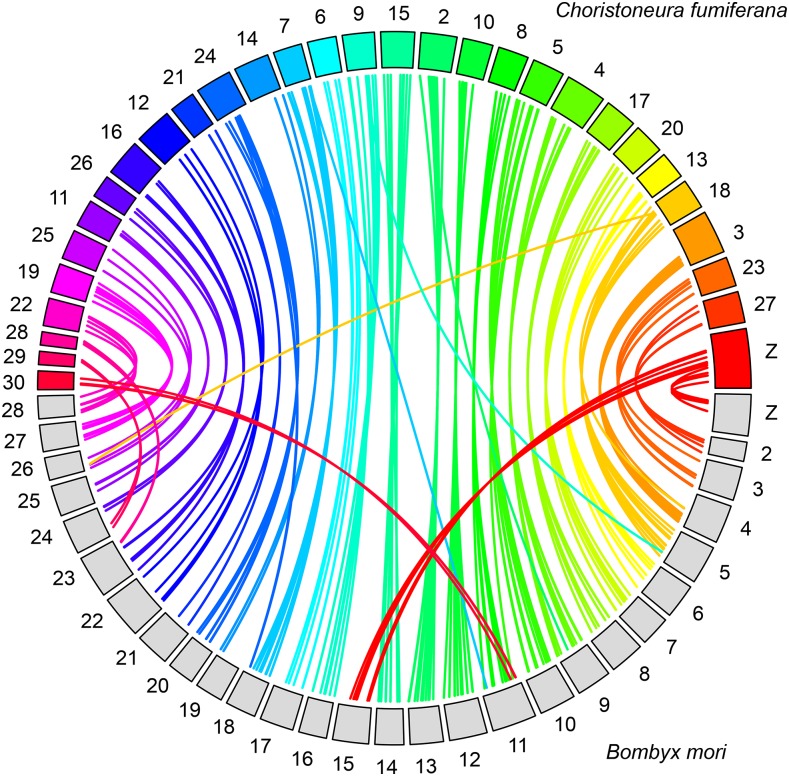
Mapping of *Choristoneura fumiferana* linkage groups onto *Bombyx mori* chromosomes. Each colored box represents one of the 30 *C. fumiferana* linkage groups (numbering from Lep-MAP output), while each gray box represents one of the 28 *B. mori* chromosomes (*B. mori* chromosome numbering; http://sgp.dna.affrc.go.jp/KAIKO). Each of the 285 connector lines identifies a one-to-one orthologous match between *C. fumiferana* and *B. mori* (TBLASTX analysis, expect value cut-off: 1.0e-03). Note that the *C. fumiferana* Z chromosome displays matches to both chromosomes Z and 15 in *B. mori*.

Among the sequences associated with the 3586 markers making up the *C. fumiferana* linkage map, 285 (7.9%) generated a significant hit in TBLASTX searches against the *B. mori* genome (expected value cut-off: 1.0e-03; Figure 5, File S2 and File S3). We inferred chromosomal homology between *C. fumiferana* and *B. mori* on the basis of 2 to 20 significant hits per chromosome. This analysis revealed one interesting feature: the Z chromosome of *C. fumiferana* corresponds to the fusion of chromosomes Z and 15 in the *B. mori* genome (Figure 5).

A similar synteny analysis targeting the *M. cinxia* genome yielded 176 significant TBLASTX hits and, overall, confirmed the above inferences about chromosome homology based on the *B. mori* genome (File S2 and File S3). However, LG 30 of *C. fumiferana* generated no significant hits in TBLASTX searches against *M. cinxia* chromosomes; in this case, chromosome homology was first inferred, and then confirmed with an earlier *M. cinxia*–*B. mori* synteny analysis (Ahola *et al.* 2014; see File S3 for details).

Given that the fusion of the Z chromosome with an autosome can significantly increase the adaptive potential of a species (Orr 2010), we conducted TBLASTX analyses against the NCBI nr database using, as queries, *C. fumiferana* sequences associated with markers located in the Z chromosome region corresponding to the *B. mori* chromosome 15 (Table 2). One Z-linked marker sequence revealed a significant match (expect value 5*10^−6^) with an ABC transporter C2 (*ABCC2*) protein of *B. mori*, which confers resistance to *Bacillus thuringiensis* toxin Cry1Ab (Atsumi *et al.* 2012).

**Table 2:** SNP markers located in the Z chromosome region corresponding to *B. mori* chromosome 15 and identified in sequences displaying significant TBLASTX hits for known proteins

Gene product	Function	Protein access. No. (organism)	No. of hits	Marker Identifier^1^	Position (cM)	E-value	Marker position on *B. mori* chromosome 15
Cap-specific mRNA (nucleoside-2’-O-)- methyltransferase transcript variant X2	Modifies 5′ end of mRNA	XM_011563421.1 (*Plutella xylostella*)	1	12	21.3	7.92*10^−7^	980069-980122
Spectrin alpha chain transcript variant 3	Molecular scaffold protein; determination of cell shape	XM_012692410.1 (*Bombyx mori*)	2	22	31.88	10*10^−6^	1657717-1657661
				23	31.88	5.07*10^−5^	1657810-1657754
Neuropathy target esterase/Swiss cheese	Membrane lipid homeostasis	XM_011570849.1 (*Plutella xylostella*)	2	44	35.16	1.82*10^−5^	2595540-2595484
				62	45.7	1.82*10^−5^	2595507-2595451
AP-3 complex subunit delta-1	Facilitates budding of vesicle from the Golgi membrane	XM_012692363.1 (*Bombyx mori*)	2	94	55.7	3.28*10^−5^	9224907-9224963
				104	56.64	3.43*10^−5^	9226413-9226357
DNA binding protein RFX5	Regulation of MHCII molecule transcription	XP_021189473.1 (*Papilio xuthus*)	1	106	56.64	5*10^−6^	9598701-9598757
Glutamate-cysteine ligase catalytic subunit	Glutathione biosynthesis	XM_004924611.2 (*Bombyx mori*)	1	110	58.55	2.7*10^−6^	11234605-11234546
Cyclic nucleotide-gated cation channel	Sensory transduction (including pheromone perception)	XM_012689102.1 (*Bombyx mori*)	2	111	58.55	2.93*10^−7^	10837125-10837066
				112	58.55	3.65*10^−6^	10837143-10837087
ABC transporter family C protein (ABCC2)	Transmembrane transport/detoxification	XM_012692465.1 (*Bombyx mori*)	1	198	62.31	5*10^−6^	11251268-11251315
Serine/threonine-protein phosphatase 2A	Removal of phosphate groups from phosphorylated Ser/Thr residues	XM_012697398 (*Bombyx mori*)	1	203	62.31	2.5*10^−5^	13143820-13143764

1 See File S2, linkage map details sheet, column named *marker identifier*.

## Discussion

We have combined cytogenetic approaches and a high-density SNP-based linkage map to explore the organization of the spruce budworm genome. Our karyotype analyses confirmed the presence of 30 pairs of chromosomes in *Choristoneura fumiferana*, a number commonly found in Tortricinae, one of three subfamilies recognized in the Tortricidae (Robinson 1971; Ennis 1976; Harvey 1997; Šíchová *et al.* 2013). Our molecular cytogenetic analyses also enabled identification of a noticeably larger pair of chromosomes in both sexes. In females, one of these two large chromosomes was strongly stained by both DAPI and fluorescently-labeled female gDNA (GISH analysis). In Lepidoptera, this staining pattern is typical of the W chromosome (Traut and Marec 1997; Mediouni *et al.* 2004; Fuková *et al.* 2005; Yoshido *et al.* 2006). As such, our findings provide evidence for the existence of a WZ/ZZ (female/male) sex chromosome system in the spruce budworm; *C. fumiferana* can now be added to the modest list of ∼90 lepidopteran species for which the sex chromosome system has been identified [F. Marec, unpublished data; Traut *et al.* (2007) and Marec *et al.* (2010) reported ∼40 species]. Interestingly, the number of lepidopteran species for which the sex chromosome system has been described is much smaller than the number for which chromosomes have been quantified (∼1,000; Robinson 1971; Ennis 1976; Blackmon *et al.* 2017). This difference may be attributed to the fact that many earlier studies used male germ cells, which are appropriate for chromosome counts, but not for identifying the sex chromosomes (Traut *et al.* 2007; Sahara *et al.* 2012). In recent years, the use of female meiotic pachytene chromosomes combined with techniques of molecular cytogenetics (FISH, CGH, GISH, etc.), has facilitated the characterization of sex chromosomes. Thus, in coming years, we can anticipate an increase in the number of species for which the sex chromosome system has been characterized (Sahara *et al.* 2012).

The ancestral chromosome number in Lepidoptera is inferred to be n = 31 (Suomalainen 1969; Lukhtanov 2000; Ahola *et al.* 2014; Yasukochi *et al.* 2016). From this ancestral state, the number of chromosomes has evolved through chromosome fission/fusion events (De Prins and Saitoh 2003; Brown *et al.* 2004; Marec *et al.* 2010). In tortricids, the reduced number of chromosome pairs relative to the ancestral state (*i.e.*, n = 28 in Olethreutinae and n = 30 in Tortricinae, instead of 31) and the presence of large sex chromosomes suggest a sex chromosome-autosome fusion event during the evolution of their karyotypes (Fuková *et al.* 2005; Šíchová *et al.* 2013). Indeed, a recent cytogenetic study confirmed that the Z chromosome in three different tortricid species (*Cydia pomonella*, *Lobesia botrana*, and *Eupoecilia ambiguella*) arose from the fusion of an ancestral Z chromosome and an autosome (Nguyen *et al.* 2013). In our study, synteny analysis comparing the 30 linkage groups of *C. fumiferana* with the 28 chromosomes comprising the *B. mori* genome provided strong evidence for the presence of the same neo-Z chromosome in the spruce budworm genome. A Z chromosome-autosome fusion can have a significant impact on the adaptive capacity of a species. Indeed, the hemizygous state of the Z-linked genes in the female Lepidoptera increases the efficiency of natural selection acting on recessive mutations (faster X/Z evolution; Charlesworth *et al.* 1987; Bachtrog *et al.* 2009). In tortricids, the Z chromosome-autosome fusion is considered to have contributed to the adaptive success and the important radiation of this taxon (Nguyen *et al.* 2013). Indeed, the part of the neo-Z chromosome derived from an autosome corresponds to chromosome 15 in *B. mori*, which is known to bear clusters of genes involved in detoxification of plant metabolites and genes conferring insecticide resistance (Nguyen *et al.* 2013), such as the *ABC transporter C2* (*ABCC2*) gene associated with recessive resistance to the *Bacillus thuringiensis* (Bt) toxin (Atsumi *et al.* 2012; Park *et al.* 2014; Xiao *et al.* 2014). In our study, the sequence of a marker located on the Z linkage group showed significant similarity (expect value: 5*10^−6^) to the ABCC2 protein located on chromosome 15 in *B. mori*. Thus, Z-linkage of the *ABCC2* gene in *C. fumiferana* provides a genomic context favorable to the selection and rapid spread of Bt resistance in spruce budworm populations. Since there is significant variation in Bt tolerance among spruce budworm populations (van Frankenhuyzen *et al.* 1995), our results stress the importance of making rational use of Bt, as it is the main insecticide used to suppress spruce budworm populations in Canadian provinces (van Frankenhuyzen 2013).

The W chromosome of *C. fumiferana* displayed distinctive features relative to other chromosomes, including the Z chromosome. Indeed, the staining patterns obtained with our FISH assays revealed a W chromosome that is thoroughly and homogeneously heterochromatinized, *i.e.*, constituted of condensed chromatin. Studies examining the composition of the heterochromatinized W chromosome in other lepidopterans have pointed to a high content of repetitive sequences, transposable elements, degenerated protein-coding genes, and sequences of mitochondrial origins (Abe *et al.* 2005; Fuková *et al.* 2007; Vítková *et al.* 2007; Traut *et al.* 2013; Lammermann *et al.* 2016). Such an unusual composition raises questions about the involvement of this chromosome in sex determination in Lepidoptera. For instance, in *Samia cynthia*, the W chromosome does not seem to be involved in sex determination (Yoshido *et al.* 2016). However, in *B. mori*, a dominant female-determining factor (*Fem*) located on the W chromosome has been shown to promote femaleness (Fujii and Shimada 2007). Recently, this feminizing factor was determined to be a small RNA (*Fem* piRNA, Kiuchi *et al.* 2014). Considering the above mentioned examples, the sex determination mechanism in the spruce budworm has a high chance of being specific to this species. In *C. fumiferana* and other pest species, identification of the sex determination mechanism has relevance beyond questions of basic evolutionary biology, as this knowledge could enable development of alternative pest management approaches based on the sterile insect technique (SIT) (Whyard *et al.* 2015; Darrington *et al.* 2017). The SIT method is based on rearing and releasing a large number of sterile males into wild populations with the aim to decrease reproductive success (Marec *et al.* 2005). RNA interference (RNAi) targeting genes involved in female sex determination has been used successfully to eliminate females during the rearing process, while RNAi could also be used to silence genes involved in spermatogenesis and induce male sterility (Whyard *et al.* 2015). SIT could thus offer an interesting alternative to the insecticide Btk (see above) to manage spruce budworm populations.

Concerning the heterochromatinization patterns of the W chromosome, those observed here in the spruce budworm are similar to patterns reported for the tortricid *Cydia pomonella* (Fuková *et al.* 2005; Šíchová *et al.* 2013). However, heterochromatinization of the W chromosome is partial in two other tortricid species, *Lobesia botrana* and *Eupoecilia ambiguella* (Šíchová *et al.* 2013). According to Šíchová *et al.* (2013), this pattern suggests that the tortricid W chromosome originated from the fusion of an ancestral W chromosome with an autosome, most likely the homolog of chromosome 15 in *B. mori*, as shown for the Z chromosome (see above). Thus, the spruce budworm appears to have a neo-W chromosome whose complete heterochromatization may have resulted from more rapid molecular degeneration of its neo-part than that affecting the W chromosomes of *L. botrana* and *E. ambiguella*.

To our knowledge, linkage maps have been published for 10 different lepidopteran species, half of which belong to the Papilionoidea superfamily (Tobler *et al.* 2004; Wang and Porter 2004, Jiggins *et al.* 2005; Kapan *et al.* 2006; Van’t Hof *et al.* 2008; Beldade *et al.* 2009; Winter and Porter 2010; The Heliconius Genome Consortium 2012; Rastas *et al.* 2013; Ahola *et al.* 2014; Li *et al.* 2015; Davey *et al.* 2016; Van Belleghem *et al.* 2017; Rastas 2017). The remaining linkage maps were developed for species belonging to different and somewhat distantly related superfamilies: Bombycoidea (Miao *et al.* 2005; Yasukochi *et al.* 2006; Yamamoto *et al.* 2008), Pyraloidea (Dopman *et al.* 2004), Yponomeutoidea (Heckel *et al.* 1999; Baxter *et al.* 2011), Geometroidea (Van’t Hof *et al.* 2013) and Noctuoidea (Cheng *et al.* 2017). In the superfamily Tortricoidea, attempts were made earlier to develop a linkage map based on 46 backcross progeny of two closely related budworms, *C. occidentalis biennis* and *C. occidentalis occidentalis* (Brunet 2014), but a limited number of SNPs (438) could be ordered and the number of linkage groups was higher (n = 51) than expected for this species (n = 30). Differences in accuracy and completeness between the *C. occidentalis* and the *C. fumiferana* linkage maps could be partly due to differences in genetic distance between the two pairs of populations used. Indeed, a recent study suggests that the genetic distance between the *C. occidentalis biennis* and *C. occidentalis occidentalis* populations sampled by Brunet (2014) was clearly lower than that between the *C. fumiferana* populations used in the present study (see Brunet *et al.* 2017). However, given that *C. fumiferana* and *C. occidentalis* are two closely related species (Dupuis *et al.* 2017), we will now be examining the possibility of combining the two data sets to further increase marker density on our linkage map.
